# Evaluation of the Synergistic Antimicrobial Activity of Essential Oils and Cecropin A Natural Peptide on Gram-Negative Bacteria

**DOI:** 10.3390/ani15020282

**Published:** 2025-01-20

**Authors:** Filippo Fratini, Chiara Pecorini, Ilaria Resci, Emma Copelotti, Francesca Paola Nocera, Basma Najar, Simone Mancini

**Affiliations:** 1 Department of Veterinary Sciences, University of Pisa, Viale delle Piagge 2, 56124 Pisa, Italy; filippo.fratini@unipi.it (F.F.); pecorinichiara@gmail.com (C.P.); ilaria.resci@phd.unipi.it (I.R.); emma.copelotti@phd.unipi.it (E.C.); simone.mancini@unipi.it (S.M.); 2 Department of Veterinary Medicine and Animal Production, University of Naples “Federico II”, Via Delpino 1, 80137 Naples, Italy; 3 RD3—Pharmacognosy, Bioanalysis & Drug Discovery Unit, Analytical Platform of the Faculty of Pharmacy, Faculty of Pharmacy, Free University of Brussels, Campus Plaine, Blvd Triomphe, CP 205/5, B-1050 Brussels, Belgium; basmanajar@hotmail.fr

**Keywords:** winter savory, cinnamon, bergamot, Cecropin A, synergy

## Abstract

In an era dominated by the phenomenon of antibiotic resistance, it is increasingly important to look for alternatives to synthetic antibiotics. In light of these considerations, the synergistic use of essential oils and Antimicrobial Peptides (AMPs) seems a viable strategy. In this study, we assessed the Minimum Inhibitory Concentration (MIC), Minimum Bactericidal Concentration (MBC) and Fractional Inhibitory Concentration (FIC) of three Essential Oils (EOs): winter savory (*Satureja montana*), bergamot (*Citrus bergamia*) and cinnamon (*Cinnamomum zeylanicum*) and of the insect antimicrobial peptide Cecropin A (CecA), alone and in combination with EOs, against two Gram-negative ATCC bacterial strains: *Escherichia coli* and *Salmonella enterica* serovar Typhimurium. The MIC results showed that winter savory EO (*Sm*EO) and cinnamon EO (*Cz*EO) exhibited the strongest antibacterial activity against both bacterial strains, whereas bergamot EO (*Cb*EO) and CecA demonstrated comparatively lower antibacterial efficacy. These results were also confirmed by the MBC values. The FIC Indices (FICI) revealed that the most effective synergies were observed with the combinations *Sm*EO/*Cz*EO and *Sm*EO/*Cb*EO against *E. coli*, while against *S. enterica* Typhimurium the best combinations were *Cb*EO/*Cz*EO and *Sm*EO/*Cz*EO. Regarding CecA, although it was not the most efficient agent either individually or in combination, it is noteworthy that, when combined, it exhibited antibacterial activity even at a 1:64 dilution.

## Simple Summary

The rise of antibiotic resistance has driven the search for new and alternative strategies to traditional antimicrobial agents. In this context, the combined use of essential oils and natural antimicrobial peptides represents a promising approach. This study aimed to evaluate the inhibitory effects of three essential oils—winter savory, bergamot, and cinnamon—and the insect-derived antimicrobial peptide Cecropin A, both individually and in combination, against two Gram-negative pathogenic bacteria (*Salmonella enterica* serovar Thyphimurium and *Escherichia coli*) as a potential weapon to counteract antimicrobial resistance. The obtained results showed a synergistic effect of essential oils and Cecropin A on both bacteria, suggesting the need for further research to explore the efficacy of natural extracts. These substances hold potential for applications not only in the medical field but also in the food and feed industries.

## 1. Introduction

The phenomenon of antibiotic resistance is now a global concern that represents one of the most important challenges facing the scientific world in this early 21st century. The excessive and sometimes reckless use of antibiotics in the medical, veterinary, agricultural and livestock sectors over the years may have increased the severity and speed of this phenomenon [1]. A new approach involving the use of natural substances that can, if not replace, at least flank antibiotics with a view to reducing their use therefore appears increasingly to be the way forward for the scientific world [2].

Essential Oils (EOs) and natural Antimicrobial Peptides (AMPs) are among the natural substances showing the greatest promise for application potential [3,4]. Several authors have reported the beneficial effects of EOs as alternatives to antimicrobial compounds in animal production [5]. The antimicrobial properties of EOs may be further enhanced by mixing different EOs or combining them with other natural compounds, such as AMPs [6,7].

Investigating their synergistic effects could be particularly beneficial, as this approach may amplify their effectiveness while allowing the use of lower doses, potentially minimizing side effects and costs [8]. Although there are several examples of synergies between EOs in the literature, there are limited studies on combinations of AMPs and even fewer on the associations between EOs and AMPs [9].

Among the EOs recognized for their strong antibacterial properties, those obtained from plants belonging to the Lamiaceae family are probably the most well-known [10]; within this group, *Satureja montana* Essential Oil (*Sm*EO) is one of the most active by virtue of its bioactive molecules. The bioactive compounds most commonly found in *Sm*EO are carvacrol, p-cymene, which is its precursor, and thymol, whose antibacterial effectiveness has long been proven by numerous scientific studies [11,12,13]. The in vitro efficacy of *Sm*EO is confirmed by many data available in the scientific literature and also by our own experience, especially in the field of veterinary medicine [14,15,16,17,18]

Another EO with well-known antimicrobial properties is *Cinnamomum zeylanicum* (*Cz*EO), whose key components responsible for its antibacterial effectiveness are undoubtedly eugenol and cinnamaldehyde [19,20].

An EO that is probably less well-known but possesses notable antimicrobial properties is bergamot oil, extracted by cold-pressing the peel of *Citrus bergamia* fruit. This extraction method retains a composition of volatile organic compounds closer to what the plant naturally produces compared to oils obtained through steam distillation [21]. Its antimicrobial activity appears to be primarily due to compounds such as limonene, linalool and linalyl acetate, which have substantial evidence supporting their effectiveness [22,23]. The scientific investigations reported as supporting bibliography also demonstrate good efficacy, at least in vitro, of these EOs against Gram-negative bacteria.

Cecropin AMPs were first isolated from the hemolymph of *Hyalophora cecropia* and characterized for their activity against Gram-positive and Gram-negative bacteria [24]. These peptides were subsequently identified in other Lepidoptera, Coleoptera and Diptera species [6]. Cecropin A is a cationic peptide that belongs to the α-helix conformation group [25]. The available scientific literature shows that Cecropin A has strong antibacterial activity against Gram-negative bacteria, an aspect usually uncommon to find in many natural substances [26].

According to the mechanism of action, AMPs were observed to induce morphological deformations of bacterial cells and the release of the cellular contents, whereas EOs tend to split and completely envelope bacteria [7].

The main goal of our investigation was to assess the antibacterial activity of winter savory, bergamot and cinnamon EOs and the peptide Cecropin A against *Escherichia coli* and *Salmonella enterica* serovar Typhimurium, bacterial species selected for their well-documented antimicrobial resistance profiles [27].

## 2. Materials and Methods

### 2.1. Bacterial Strains

The American Type Culture Collection (ATCC) strains *Escherichia coli* ATCC 15325 and *Salmonella enterica* serovar Typhimurium ATCC 14028 were employed in the study.

Both strains were stored at −80 °C in a glycerol suspension until use. Before MIC and FIC determinations, the strains were cultured in BHI (Brain Hearth Infusion, Oxoid, Milan, Italy) broth for 24 h at 37 °C in aerobic conditions.

### 2.2. Essential Oils and Peptide Employed

For this study, three different essential oils of *Satureja montana* L., *Cinnamomum zeylanicum* and *Citrus bergamia* Risso were tested based on their previously reported potential antimicrobial properties [14,16,28,29]. The EOs used for the tests were purchased directly on the market (FLORA^®^, Pisa, Italy) and, according to the product leaflet, were obtained by steam distillation from *Satureja montana* L. and *Cinnamomum zeylanicum* Blume and by cold pressing of the peel of *Citrus bergamia* Risso.

The analysis was conducted using a Varian CP-3800 gas chromatograph equipped with a DB-5 capillary column and a Varian Saturn 2000 ion trap mass detector (SpectraLab Scientific Inc., Markham, ON, Canada). The conditions were as follows: oven temperature programmed from 60 °C to 240 °C at 3 °C/min, injection temperature set to 220 °C and transfer line temperature at 240 °C. Helium served as the carrier gas at a flow rate of 1 mL/min. Compound identification was based on retention time comparison with authentic standards, and mass spectra were matched against commercial libraries (NIST 98, ADAMS) and a custom library (Table 1).

**Table 1:** Chemical composition of the essential oils of *Citrus bergamia* (*Cb*), *Cinnamomum zeylanicum* (*Cz*) and *Satureja montana* (*Sm*).

Compounds	LRI	*Cb*	*Cz*	*Sm*
α-thujene	932	0.3	0.2	0.5
α-pinene	940	1.1	0.8	1.1
camphene	955		0.3	0.6
benzaldheyde	965		0.3	
sabinene	978	1.1		
β-pinene	981	6	0.3	0.6
1-octen-3-ol	982			0.2
3-octanone	987			0.1
myrcene	993	0.9		1.4
α-phellandrene	1006		1.1	0.2
α-terpinene	1019		0.6	1.5
p-cymene	1026	0.4		13.7
o-cymene	1026		2.5	
limonene	1032	30.8		0.4
β-phellandrene	1033		4.4	
(Z)-β-ocimene	1042			0.1
(E)-β-ocimene	1053	0.2		0.1
γ-terpinene	1062	7.4		8.7
cis-sabinene hydrate	1072			0.3
p-mentha-2,4(8)-diene	1088		0.1	
terpinolene	1090	0.2		0.2
Linalool	1102	12.5	5	1.3
camphor	1148			0.2
4-terpineol	1180		0.4	3
α-terpineol	1192	0.2	0.7	0.8
cis-dihydrocarvone	1193			0.1
1-octanyl acetate	1214	0.2		
(Z)-cinnamaldehyde	1223		1.2	
(Z)-ocimenone	1229		0.1	
methyl thymol	1235			0.1
Neral	1242	0.2		
methyl carvacrol	1245			4.2
linalool acetate	1260	34.9		
(E)-cinnamaldehyde	1274		55	
geranial	1276	0.2		
bornyl acetate	1289			0.1
Thymol	1290			5.8
carvacrol	1301			48
α-terpinyl acetate	1352	0.3		
Eugenol	1361		4.6	
neryl acetate	1368	0.5		
α-copaene	1376		1.6	
geranyl acetate	1386	0.4		
β-caryophyllene	1418	0.5	10.2	3.9
trans-α-bergamotene	1437	0.5		
aromadendrene	1441			0.2
(E)-cinnamyl acetate	1449		1.1	
α-humulene	1456		2.8	0.2
(E)-β-farnesene	1460	0.1		
γ-muurolene	1477			0.1
viridiflorene	1495			0.2
β-bisabolene	1509	0.7		0.7
trans-γ-cadinene	1513			0.2
δ-cadinene	1523			0.3
caryophyllene oxide	1582		1.4	0.6
humulene epoxide II	1607		0.2	
tetradecanal	1612		0.1	
benzyl benzoate	1766		1.8	
bergaptene	2057	0.1		

The peptide used for the tests was Cecropin A (CecA) (C184H313N53O46), whose mechanism of action is mainly explicated by attacking the membrane and changing its permeability [30,31,32]. Cecropin A was purchased directly from the market (ISCA BIOCHEMICALS^®^, Exeter, UK).

According to the manufacturer’s information in the package insert, the purity of the peptide was considered to be 97% and was determined by High Performance Liquid Chromatography (HPLC). Its appearance was that of a white, impalpable powder, which had to be stored in the dark and at refrigeration temperatures after reconstitution in dimethyl sulfoxide (DMSO) at a rate of 1 mg/mL. After that, a work solution stock was prepared with Cecropin A at the concentration of 64 μg/mL, diluted in DMSO.

### 2.3. Determination of Minimum Inhibitory Concentration (MIC)

EOs MIC values were determined using a two-fold serial microdilution method according to Wiegand et al. [33], with slight modifications reported by Fratini et al. [34]. Briefly, 190 µL of BHI was distributed in each well of a 96-well polypropylene microtiter plate (Biosigma S.r.l. a Dominique Dutscher Company, Venice, Italy), except for the first column. EO dilution was prepared as 1 part of EO, 3 parts of DMSO (dimethyl sulfoxide (Carlo Erba, Milan, Italy) and 4 parts of BHI (Oxoid, Milan, Italy), to a final ratio of 1:3:4 (*v*/*v*). As for Cecropin A, a direct 1:2 dilution (32 μg/mL) in BHI of the peptide reconstituted in sterile distilled water was performed in the first well of the row. Then, 380 µL of EO or Cecropin A dilution was dispensed in the first well of each strain row, and two-fold dilution series were performed on the 10th well. From the 10th wells of each row, 190 µL were discarded. Then, 10 µL of a bacterial suspension adjusted to 0.5 of the McFarland standard turbidity scale was added to each well of the respective strain row to reach a final volume of 200 µL. The 11th and 12th wells were employed for the positive and negative controls, respectively. The positive control was composed of bacterial inoculum and broth without EOs, while the negative control consisted only of broth. Microplates were incubated at 37 ◦C for 24 h in a humid chamber. EOs and peptide MIC determinations were performed in triplicate, and for each strain the values were expressed as mode.

### 2.4. Determination of Fractional Inhibitory Concentration (FIC)

The combined effect of EOs or of an EO with Cecropin A (FIC) was evaluated by the microdilution chequerboard method, with some modifications according to Fratini et al. [34], in which the pivotal novelty of the purpose is the simultaneous determination of the MIC and FIC values. Assays were performed on 96-well polypropylene microtiter plates. FIC determinations were performed in triplicate. For each replicate, FIC Indices (FICI) were calculated using the following formula:

FICI = FIC _first substance_ + FIC _second substance_ (1)

FIC value was calculated using the following formula:

FIC_n_= MIC_n_ in combination/MIC_n_ alone (2)

According to the FICI interpretation model of Fratini et al. [34], a Synergistic Effect (SynE) is detected when FICI value < 1; a Commutative Effect (ComE) when FICI value = 1; an Indifferent Effect (IndE) when 1 < FICI value ≤ 2 and an Antagonistic Effect (AntE) when FICI value > 2. The bar plots were created with Rstudio version 4.2.1. using packages *ggplot2*, *tidyr*, *dplyr*, *RcolorBrewer*, *ggpubr* [35,36,37].

### 2.5. Determination of Minimum Bactericidal Concentration (MBC)

For every plate used to evaluate the FIC and MIC of each substance, a loop of material was collected from all wells showing no visible bacterial growth. Seeding was then performed on Tryptone Soy Agar (TSA) plates (Oxoid, Milan, Italy) and incubated at 37 °C for 24 h [38,39]. After the overnight incubation, any bacterial growth was assessed by comparing the obtained MIC and FIC values, allowing the evaluation of the steps between dilutions.

## 3. Results

This study aimed to evaluate the inhibitory and bactericidal activities of three types of Essential Oil (EO) and the peptide Cecropin A, both individually and in combination. Initial Minimum Inhibitory Concentration (MIC) determinations for each EO and Cecropin A revealed that *Sm*EO exhibited the lowest MIC value (1:2048) against both *E. coli* and *S.* Typhimurium. Against *E. coli*, the next lowest MIC values were observed for *CzEO* (1:512), *CbEO* (1:32) and Cecropin A (1:4), while for *S*. Typhimurium the order was *CzEO* (1:512), *CbEO* (1:16) and Cecropin A (1:4).

However, the MBC values were identical for both *E. coli* and *S.* Typhimurium. In this case, the lowest MBC values were observed for *Cz*EO (1:512) and *Sm*EO (1:512), followed by *Cb*EO (1:16) and Cecropin A (1:2). All MIC and MBC values are listed in Table 2.

**Table 2:** MIC and MBC mode values for each EO and Cecropin A determined for both bacterial strains tested.

Essential Oils and Peptide	*S.* Typhimurium ATCC 14028		*E. coli* ATCC 15325	
	MIC	MBC	MIC	MBC
*Cb*EO	1:16	1:16	1:32	1:16
*Cz*EO	1:512	1:512	1:512	1:512
*Sm*EO	1:2048	1:512	1:2048	1:512
CecA	1:4	1:2	1:4	1:2

MIC: Minimum Inhibitory Concentration; MBC: Minimum Bactericidal Concentration; *Cb*EO: *Citrus bergamia* Essential Oil; *Cz*EO: *Cinnamomum zeylanicum* Essential Oil; *Sm*EO: *Satureja montana* Essential Oil; CecA: Cecropin A.

**Figure 1:**
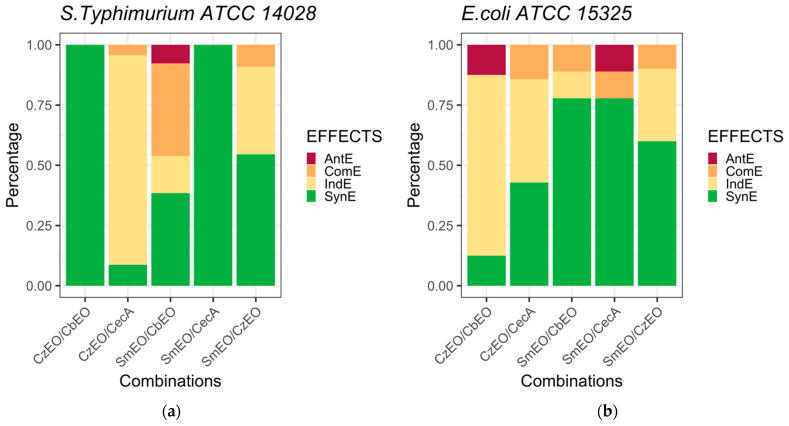
(**a**) Graphical representation of the different trend percentages of each effect for *S*. Typhimurium ATCC 14028 strain. (**b**) Graphical representation of the different trend percentages of each effect for *E. coli* ATCC 15325 strain. AntE: Antagonistic Effect; ComE: Commutative Effect; IndE: Indifferent Effect; SynE: Synergistic Effect. *Cb*EO: *Citrus bergamia* Essential Oil; *Cz*EO: *Cinnamomum zeylanicum* Essential Oil; *Sm*EO: *Satureja montana* Essential Oil; CecA: Cecropin A.

Interpretation of the model revealed that 100% Synergistic Effects (SynE) against *S.* Typhimurium were observed exclusively for the *Cz*EO/*Cb*EO and *Sm*EO/CecA combinations. No other effects were detected for these combinations, with all FICI value less than one (Table 3). For all other combinations, Commutative Effects (ComE) and Indifferent Effects (IndE) were observed (Table 4, Figure 1), with an additional 25% Antagonistic Effect (AntE) specifically for the *Sm*EO/*Cb*EO combination.

Regarding *E. coli*, none of the EO/EO combinations exhibited a 100% synergistic effect (SynE) (Table 4). However, a maximum of 77.8% synergy was observed for both the *Sm*EO/*Cb*EO and *Cz*EO/CecA combinations. Commutative Effects (ComE) and Indifferent Effects (IndE) were detected for nearly all combinations, with an Antagonistic Effect (AntE) of 12.5% and 11.1% observed for the *Sm*EO/*Cz*EO and *Cz*EO/CecA combinations, respectively. All FIC and FICI values for each combination are shown in Table 3. A complete overview of the percentage values for each effect is provided in Table 4, and a graphical representation is shown in Figure 1.

**Table 3:** FIC and FICI mode values for each EO/EO and EO/peptide combination.

Bacterial Strains	FIC_Sm_	FIC_Cz_	FICI_Sm-Cz_	FIC_Cb_	FIC_Cz_	FICI_Cb-Cz_	FIC_Cb_	FIC_Sm_	FICI_Cb-Sm_	FIC_Cz_	FIC_CecA_	FICI_Cz-CecA_	FIC_Sm_	FIC_CecA_	FICI_Sm-CecA_
	1:2048	1:8192	0.6250	1:512	1:1024	0.5313	1:32	1:8192	0.7500	1:8192	1:16	0.6250	1:16384	1:16	0.5625
	1:2048	1:4096	0.6250	1:256	1:1024	0.5625	1:32	1:4096	1.0000	1:4096	1:16	0.7500	1:2048	1:64	0.6250
	1:8192	1:1024	0.6250	1:32	1:4096	0.6250	1:512	1:2048	1.0313	1:2048	1:16	1.0000	1:8192	1:16	0.6250
	1:2048	1:4096	0.7500	1:128	1:1024	0.6250	1:256	1:2048	1.0625	1:1024	1:64	1.1250	1:2048	1:32	0.7500
*S.* Typhimurium	1:2048	1:2048	0.7500	1:32	1:2048	0.7500	1:256	1:2048	1.0625	1:1024	1:32	1.2500	1:4096	1:16	0.7500
ATCC 14028	1:4096	1:1024	0.7500	1:64	1:1024	0.7500	1:32	1:2048	1.5000						
	1:2048	1:2048	1.0000				1:128	1:1024	2.1250						
	1:32768	1:1024	1.0313				1:64	1:1024	2.2500						
	1:16384	1:1024	1.0625												
	1:8192	1:1024	1.1250												
	1:4096	1:1024	1.2500												
	1:8192	1:2048	0.3750	1:512	1:1024	0.6250	1:512	1:1024	0.5313	1:1024	1:64	0.5625	1:2048	1:64	0.5625
	1:4096	1:2048	0.5000	1:256	1:1024	1.1250	1:32	1:8192	0.5625	1:1024	1:32	0.6250	1:16384	1:8	0.5625
	1:32768	1:1024	0.5625	1:32	1:4096	1.2500	1:256	1:1024	0.5625	1:1024	1:16	0.7500	1:16384	1:8	0.5625
	1:16384	1:1024	0.6250	1:128	1:1024	1.2500	1:32	1:4096	0.6250	1:1024	1:8	1.0000	1:2048	1:32	0.6250
*E. coli*	1:2048	1:4096	0.6250	1:128	1:1024	1.2500	1:128	1:1024	0.6250	1:8192	1:4	1.0625	1:8192	1:8	0.6250
ATCC 15325	1:8192	1:1024	0.7500	1:32	1:2048	1.5000	1:128	1:1024	0.6250	1:4096	1:4	1.1250	1:2048	1:16	0.7500
	1:4096	1:1024	1.0000	1:32	1:1024	2.0000	1:32	1:2048	0.7500	1:2048	1:4	1.2500	1:4096	1:8	0.7500
	1:2048	1:8192	1.0625	1:64	1:512	2.5000	1:32	1:1024	1.0000				1:16384	1:4	1.0625
	1:2048	1:4096	1.1250				1:64	1:512	1.2500				1:32768	1:2	2.0313
	1:2048	1:2048	1.2500												
	1:4096	1:1024	1.2500												

FIC: Fractional Inhibitory Concentration; FICI: Fractional Inhibitory Concentration Index; *Sm*: *Satureja montana*; *Cz*: *Cinnamomum zeylanicum*; *Cb*: *Citrus bergamia*; CecA: Cecropin A; Columns in “grey” refers to FICI values.

**Table 4:** Determination of Synergistic Effect (SynE), Commutative Effect (ComE), Indifferent Effect (IndE) and Antagonistic Effect (AntE) for each tested EO/EO EO/CeCA combination against *S.* Typhimurium and *E. coli*.

	***S.* Typhimurium ATCC 14028**			
EO combinations *	**SynE (%)**	**ComE (%)**	**IndE (%)**	**AntE (%)**
*Cz*EO/*Cb*EO	100.00	0.00	0.00	0.00
*Sm*EO/*Cb*EO	12.50	12.50	50.00	25.00
*Sm*EO/*Cz*EO	54.55	9.09	36.36	0.00
*Cz*EO/CecA	40.00	20.00	40.00	0.00
*Sm*EO/CecA	100.00	0.00	0.00	0.00
	***E. coli* ATCC 15325**			
EO combinations *	**SynE (%)**	**ComE (%)**	**IndE (%)**	**AntE (%)**
*Cz*EO/*Cb*EO	12.5	0.00	75.00	12.5
*Sm*EO/*Cb*EO	77.78	11.11	11.11	0.00
*Sm*EO/*Cz*EO	60.00	10.00	30.00	0.00
*Cz*EO/CecA	42.86	14.28	42.86	0.00
*Sm*EO/CecA	77.78	11.11	0.00	11.11

* Interpretation model of obtained effect (%) for each EO/EO and EO/CecA combination. *Cb*EO: *Citrus bergamia* Essential Oil; *Cz*EO: *Cinnamomum zeylanicum* Essential Oil; *Sm*EO: *Satureja montana* Essential Oil; CecA: Cecropin A.

## 4. Discussion

Analyzing the MIC values, *Sm*EO highlighted the best results, showing good antibacterial activity against both *E. coli* (1:2048) and *S.* Typhimurium (1:2048). The essential oil with the lowest antibacterial activity was *Cb*EO, which inhibited the bacterial growth at dilutions of 1:32 for *E. coli* and 1:16 for *S.* Typhimurium. These results were in line with results by Lv et al. [40], who highlighted a weak antibacterial activity of *Cb*EO against *E. coli*. Similarly, Mancuso et al. [41] reported that *E.coli* was resistant to many of the EOs tested, including bergamot essential oil.

Cecropin A did not show particularly encouraging results, having inhibited *E. coli* and *S.* Typhimurium only at a dilution of 1:4, corresponding to 16 μg/mL. Of course, this is a small quantity but still considerably remarkable, considering the high peptide production costs. The MBC values generally confirm the results obtained from MIC tests, since at most they differed only by one dilution.

FIC value determinations exhibited interesting results, especially regarding the interactions between essential oils. The strongest synergistic activity was observed with the association of *Cb*EO/*Cz*EO against *S.* Typhimurium (100% FICI < 1). This finding is particularly notable given that the *Cb*EO MIC on its own did not provide values comparable to the other two tested oils. In fact, *Cb*EO alone inhibited bacterial growth only at 1:16, while in synergy with *Cz*EO, a dilution of 1:512 was enough.

Barbosa et al. [29], in 2015, reported that *Cz*EO had the highest antibacterial activity against *E. coli* and *S*. Typhimurium when compared with other tested labiate EOs. In the same study, they also evaluated the antimicrobial activity of *Cz*EO in synergy with *Rosmarinus officinalis* EO, achieving excellent results in the inhibition of *E. coli* [29]. The antibacterial activity of *Cz*EO against *E. coli* and *S*. Typhimurium is also reported by other authors, such as Singh et al. [33], Lopez et al. [34] and Raj et al. [42,43,44]. Among the best results, the combination *Cb*EO/*Sm*EO was found to be effective in inhibiting *E. coli* at dilutions of 1:512 and 1:1024, respectively. In addition, 77.78% of FICI resulted <1, showing a marked synergy between the two oils. Another noteworthy result was the synergistic activity of *Sm*EO/*Cz*EO against both bacterial strains; in fact, this blend inhibited the growth of both *E. coli* and *S.* Typhimurium, even at dilutions of 1:32768. In detail, *Sm*EO inhibited *E. coli* growth at dilutions of 1:32768 when combined with *Cz*EO at 1:1024.

Vitanza et al. [28] showed an antimicrobial activity of commercial *Satureja montana* essential oil against clinical and reference Gram-positive and Gram-negative strains. They also reported synergistic interaction with gentamicin against bacterial strains and the reduction of bacterial biofilm. Similarly, Penalver et al. [45], in 2005, evaluated the antibacterial activity of some labiate EOs, such as *Thymus zygis*, *Satureja montana* and *Origanum vulgare* EOs against *E. coli* and *S*. Typhimurium; all the tested oils proved to be more active against the first microorganism. However, *Sm*EO reported the worst results, and this seemed to be due to its chemical composition, characterized by a high thymol concentration (36.56%) compared to *Origanum vulgare* (0%). Furthermore, *Sm*EO used in association with another EO with a similar chemical composition (obtained by *Thymus zygis*) showed a synergistic activity in the inhibition of *E. coli* and *S*. Typhimurium.

Similar results were obtained in the study conducted by Fratini et al. in 2014, in which was reported a good inhibition activity of *Sm*EO against *E. coli. Sm*EO was also tested in synergy with *Thymus vulgaris* ct. Thymol EO, providing better results than those obtained individually for each compound [14]. The Cecropin A MIC alone showed markedly lower results (1:4) than the other essential oils; a strong synergistic activity was shown in the FIC tests against *S.* Typhimurium and *E. coli*. For both bacterial strains, Cecropin A was already active at dilutions of 1:64, corresponding to 1 μg/mL. In addition, 77.78% (for *E. coli*) and 100% (for *S*. Typhimurium) of FICI resulted <1, highlighting a noteworthy synergy between the two compounds. The blend *Sm*EO/Cecropin A demonstrated remarkable effectiveness against both bacterial strains; in particular, *Sm*EO proved to be active against both bacterial strains at dilutions of 1:2048 when combined with Cecropin A at 1:64.

There are not many studies in which *Cb*EO is employed alone against Gram-negative bacterial strains, and this could be justified by the fact that the antibacterial activity of this oil on its own does not seem to be very effective. Our results, however, open the way for new discussions on the use of *Cb*EO in synergy with other compounds represented in several EOs, considering that *Cb*EO with *Sm*EO and *Cz*EO was much more active than when individually used. In particular, it would be desirable to study the interactions of the major chemical compounds present in the oils, although it has been pointed out that minority components often play a key role in synergy effects [14]. Regarding the synergistic activity evaluation of the oils reported in the present investigation, there are still no similar studies available in the literature that allow us to compare the obtained results. Although our results are generally in line with those in the literature, several factors should be considered that could account for differences in the EOs antimicrobial activity, often leading to contrasting results, even when the same substances are used. For example, these factors include the different structure of Gram-negative and Gram-positive bacteria, the chemical composition and concentration of each tested oil, variations in plant growth conditions and the oil extraction process.

Chemical composition plays an important role, as it directly interferes with the antimicrobial activity. In fact, oils with higher content of terpenoids, terpenes or related compounds tend to be more active, which could explain why, based on our results, *Sm*EO and *Cz*EO were the most effective [29,46]. Discussing the results, it is also worth highlighting that Gram-negative bacteria, during in vitro tests, are usually less susceptible to the antibacterial activity of essential oils, since they have the outer part of the cell wall composed of Lipopolysaccharides (LPS), which provide remarkable resistance and prevent the diffusion and accumulation of EOs in the bacterial cell [47].

The two N-terminal residues of Cecropin A are crucial for the interaction with negatively charged bacterial cell membranes [48]. Also, cecropin A has been proven to be effective against planktonic and sessile biofilm-forming UPEC cells [49].

Regarding the tested peptide, to the best of our knowledge, there is only one study in the literature that compares to our results. Li and Gray, in 2003, tested Cecropin A and B and magainins 1 and 2 against *E. coli*. Both cecropins inhibited bacterial growth at a concentration of 2 μg/mL, while no antibacterial activity was detected for magainins [50]. Concerning *E. coli*, Lyu et al. [43], in 2016, showed that melittin is able to inhibit this microorganism growth, even at concentrations of 6 μg/mL [51]. Romanelli et al. [44], in 2011, observed temporins antibacterial action only at high concentrations (>100 μg/mL), whereas jelleins, usually endowed with antibacterial efficacy against many microorganisms, especially Gram-positive ones, proved to be inactive. These results confirm those obtained by Rosenfeld in 2006 [52,53].

Most of the studies concerning the antimicrobial activity of peptides in synergy with EOs use bacterial or fungal peptides such as nisine, vancomycin or amphotericin B [54,55,56].

No other study in the bibliography tested the antimicrobial activity of Cecropin A with EOs of common use, such as *Cz*EO or *Sm*EO. Although in our investigation Cecropin A MIC is higher than the one reported in the only study present in the literature, it is important to emphasize that Cecropin A, in combination with *Sm*EO and *Cz*EO, inhibited the growth of *E. coli* and *S.* Typhimurium at much lower concentrations than those found in the bibliography (1 μg/mL vs. 2 μg/mL) reported by Li and Gray [50]. The Cecropin A activity was tested in vivo on mouse models, showing its antitumoral and antiseptic activity. The Cecropin A effect has promising effects; however, due to their sensitivity to enzymatic degradation and the elevated costs for large-scale production, its use is not yet widespread [6].

## 5. Conclusions

In conclusion, our results highlight the potential antimicrobial activity of essential oils when combined with the Cecropin A peptide, particularly in combination with winter savory essential oil. This finding underscores their potential as alternative or complementary compounds in antimicrobial applications. However, further studies are required to evaluate the cytotoxic activity of these compounds at various concentrations, considering that the production cost of Cecropin A remains very high for large-scale production. Additionally, their efficacy should be assessed in living organisms.

Other potential applications include their use as additives in the food industry. Nevertheless, further research is necessary to determine whether or not incorporating these compounds would affect the properties of the final product. The present study clearly demonstrated that compounds with low antimicrobial activity on their own can increase their effectiveness when used in synergy with other natural compounds.

To sum up, the use of peptides and essential oils could be a valid alternative to limit the ever-increasing problem of antibiotic resistance in veterinary and human medicine.

## Data Availability

The data represents the original findings of this study and are available within the article.
